# Mycobacterium Simiae Infection in a Person With Cystic Fibrosis

**DOI:** 10.7759/cureus.11062

**Published:** 2020-10-20

**Authors:** Thomas S FitzMaurice, Dilip Nazareth, Caroline McCann, Martin Walshaw

**Affiliations:** 1 Adult Cystic Fibrosis Unit, Liverpool Heart and Chest Hospital NHS Foundation Trust, Liverpool, GBR; 2 Radiology, Liverpool Heart and Chest Hospital NHS Foundation Trust, Liverpool, GBR

**Keywords:** cystic fibrosis, mycobacterium, mycobacterial infection

## Abstract

Although non-tuberculous mycobacteria can be found in the airways of people with cystic fibrosis (pwCF), their role as pathogens may be uncertain, and treatment is problematic. We report the first case of *Mycobacterium simiae (*M. *simiae)*, often associated with asymptomatic disease or colonisation, which caused pulmonary infection requiring treatment in a pwCF. This report shows that M. *simiae*, rare in pwCF in the United Kingdom, can cause significant illness and highlights the diagnostic difficulty in individuals with positive smear mycobacteria that can be mistaken initially for pulmonary tuberculosis (TB).

## Introduction

The improving survival in people with cystic fibrosis (pwCF), coupled with enhanced microbiological methodology and the propensity of adults with cystic fibrosis (CF) to travel [1], has resulted in an increase in pulmonary infection and colonisation with exotic bacteria in this population [2]. These include non-tuberculous mycobacterial (NTM) species, the significance of which is uncertain for many patients and where treatment, if necessary, can be problematic [3]. We report a case of M. simiae infection requiring treatment in a UK pwCF, and the diagnostic difficulties associated with this diagnosis.

## Case presentation

A 26-year-old Caucasian male (Forced Expiratory Volume in 1 second [FEV_1_] 4.2L, 90% predicted) with CF (p.Gly551Asp/p.Arg 17Leu26) returned to the clinic in an unwell state with haemoptysis, dyspnoea and five kg weight loss over two months, having travelled widely in South East Asia for nine months. He had previously undergone thyroidectomy and ^131^I (Radioactive iodine) treatment for papillary thyroid carcinoma. A chest radiograph did not demonstrate any new changes. However, sputum was smear-positive for Mycobacterium sp., raising the possibility of pulmonary tuberculosis (TB), and he was commenced on standard quadruple therapy isoniazid (H), rifampin (R), pyrazinamide (Z), and ethambutol (E) (HRZE). However, polymerase chain reaction (PCR) for TB was subsequently negative, his symptoms settled, and at four weeks TB treatment was stopped.

**Figure 1:**
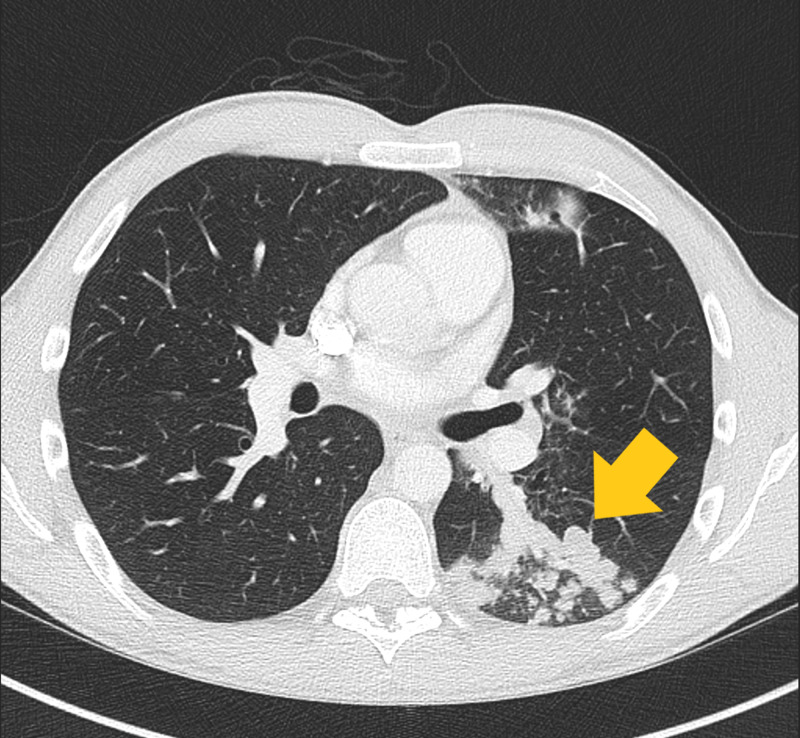
Axial view of the superior left lower lobe, demonstrating plugging of the dilated bronchial tree with tree-in-bud appearance.

**Figure 2:**
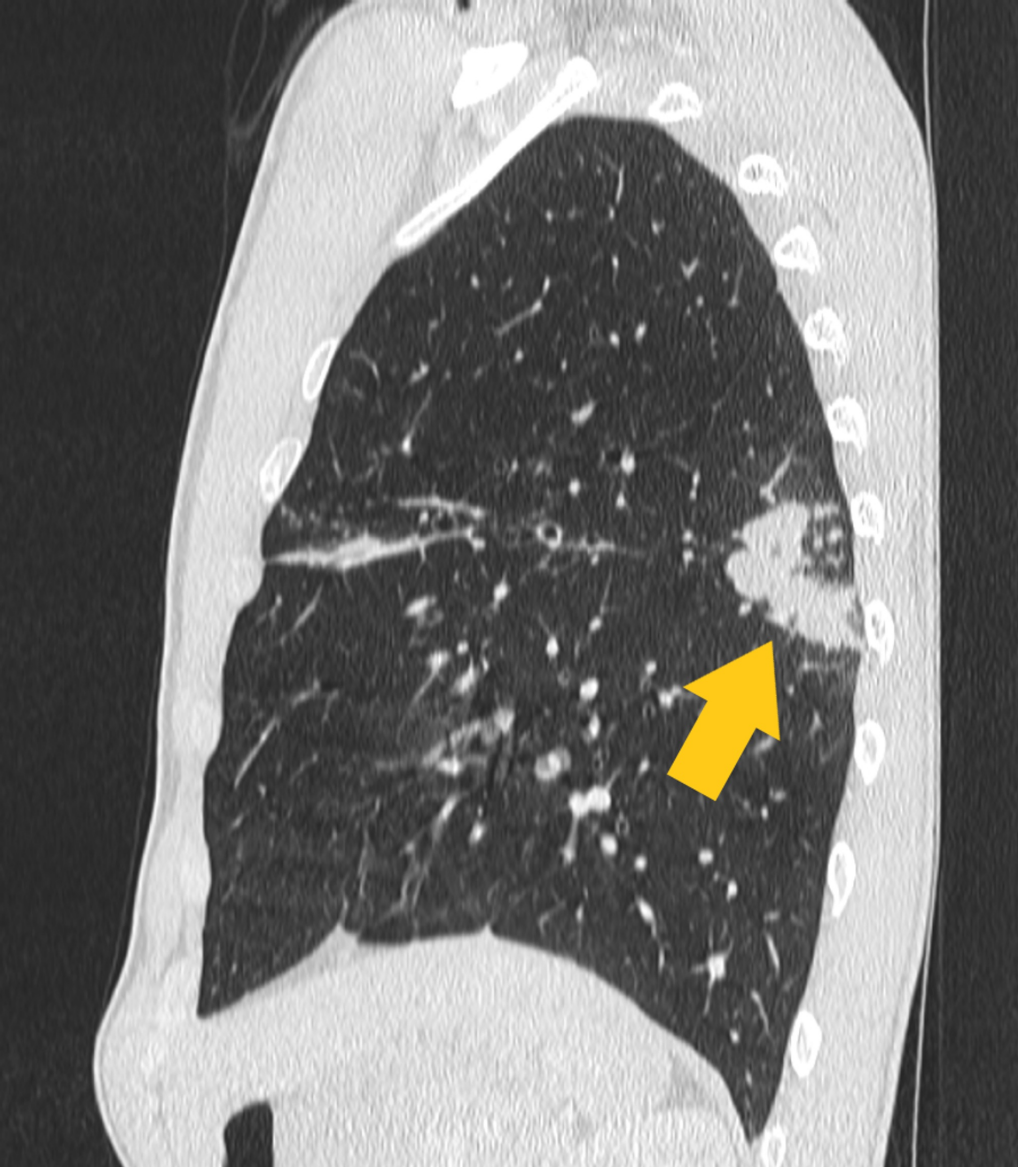
Sagittal view of the superior left lower lobe, demonstrating plugging of the dilated bronchial tree with a tree-in-bud appearance.

After 6 weeks of culture, the organism was identified as M. simiae, sensitive to clarithromycin/erythromycin but resistant to HRZE. Four weeks later he re-presented with haemoptysis, a fall in lung function (FEV_1_ from 84% to 75% predicted) and M. simiae was cultured from his sputum once again. In addition to CF-related bronchiectasis, a thoracic CT scan demonstrated new nodular infiltration in the right upper lobe and plugging of the bronchial tree with a tree-in-bud change in the left lower lobe Figures 1 and 2.

He now met American Thoracic Society/ Infectious Diseases Society of America (ATS/IDSA) diagnostic criteria for NTM treatment (two positive sputum cultures, pulmonary symptoms and CT findings, and exclusion of other likely diagnoses) as recommended by the 2015 Cystic Fibrosis Foundation and the European Cystic Fibrosis Society (CFF/ECFS) consensus guideline [4]. He was commenced on moxifloxacin (400 mg OD), co-trimoxazole (960 mg BD) and azithromycin (500mg OD). However, he developed hallucinations and abnormal dreams and moxifloxacin were withdrawn. He received 24 months of co-trimoxazole, azithromycin and ethambutol, in addition to his usual CF medications, and remains symptom-free.

## Discussion

M. simiae is a member of the Mycobacterium simiae complex, which compromises some 19 species. Infection with M. simiae was first reported in rhesus monkeys and has since been isolated in many countries, notably Cuba and the Middle East, with growing incidences in Europe and South East Asia [5]. It may be acquired from water [6] as well as nosocomial sources [7], and antibiotic resistance, as was present in this case, is common.

M. simiae is a slow-growing organism; as few as 9% of M. simiae isolates may be clinically relevant [3], and extra-pulmonary manifestations appear to be restricted to immune-compromised hosts. Our subject had a history of treated thyroid cancer, but no other known illnesses. In one study of 97 cases of M. simiae, tree-in-bud lesions were found in 50.5% of cases and consolidation in 30.9% [8], as they were in our case, versus cavitary lesions (25.8%), which are more commonly found in Mycobacterium avium complex infections.

This report has shown that whilst frequently identified as an organism of varying significance to health, M. simiae has the potential to cause significant illness in pwCF, and demonstrates the diagnostic difficulty in individuals with positive smear mycobacteria that can be mistaken initially for pulmonary TB.

## Conclusions

Whilst rare, M. simiae can cause pulmonary infection requiring treatment in people with CF. In symptomatic cases of the returning traveller, as described in this 26-year-old male with CF, M. simiae should be considered a potential pathological, rather than commensal, microbe. M. simiae may be acquired from nosocomial sources, and a thorough history, including travel, should be taken to ensure identification of the source of infection. No standard criteria for deciding whom to treat among those with CF exist, and the decision to treat should be multi-disciplinary and individualised to the patient.
